# Dietary index for gut microbiota is inversely associated with colorectal cancer risk: a case–control study

**DOI:** 10.3389/fnut.2026.1762018

**Published:** 2026-03-06

**Authors:** Qingzhen Song, Jing Lian, Yongjing Chen, Chuchu Shi, Peng Bu

**Affiliations:** 1 Department of General Internal Medicine, Shanxi Province Cancer Hospital/ Shanxi Hospital Affiliated to Cancer Hospital, Chinese Academy of Medical Sciences/Cancer Hospital Affiliated to Shanxi Medical University, Taiyuan, Shanxi Province, China; 2 Department of Pathology, Shanxi Province Cancer Hospital/ Shanxi Hospital Affiliated to Cancer Hospital, Chinese Academy of Medical Sciences/Cancer Hospital Affiliated to Shanxi Medical University, Taiyuan, Shanxi Province, China; 3 Department of Gastroenterology, Shanxi Province Cancer Hospital/ Shanxi Hospital Affiliated to Cancer Hospital, Chinese Academy of Medical Sciences/Cancer Hospital Affiliated to Shanxi Medical University, Taiyuan, Shanxi Province, China

**Keywords:** colorectal cancer, dietary index for gut microbiota, frailty, gut microbiota, inflammation, Zonulin

## Abstract

**Background:**

Gut microbial dysbiosis is a recognized contributor to colorectal cancer (CRC) development, with diet serving as a primary modifiable factor influencing microbiota composition. While previous research has largely focused on individual nutrients or the inflammatory potential of diet, few studies have investigated dietary patterns explicitly designed to support gut microbiota health in relation to CRC risk.

**Methods:**

In this case–control study, 350 adults (175 newly diagnosed CRC patients and 175 age- and sex-matched controls) were recruited. Dietary intake was assessed using multiple 24-h recalls and a validated food-frequency questionnaire to calculate the Dietary Index for Gut Microbiota (DI-GM), which emphasizes prebiotic fibers, polyphenols, fermented foods, fruits, vegetables, legumes, and whole grains, while penalizing ultra-processed and pro-inflammatory foods. Anthropometric, lifestyle, inflammatory (CRP, IL-6), frailty (mFI-5), intestinal permeability, and psychological indicators were measured. Logistic regression estimated CRC odds across DI-GM tertiles, adjusting for potential confounders.

**Results:**

CRC patients had significantly lower DI-GM scores than controls (7.29 ± 2.70 vs. 11.34 ± 2.55; *p* < 0.001). Higher DI-GM scores were associated with lower systemic inflammation, lower frailty, and fewer depressive and sleep-related symptoms. Individuals in the highest DI-GM tertile had 68% lower odds of CRC compared with the lowest (OR = 0.32; 95% CI 0.19–0.55; P-trend < 0.001).

**Conclusion:**

Greater adherence to a gut microbiota-supportive dietary index is independently associated with a lower risk of colorectal cancer, as well as with more favorable profiles of systemic inflammation, gut barrier integrity, and psychosocial health. These findings highlight the potential of microbiota-targeted dietary strategies for CRC prevention and support the need for future prospective and interventional research.

## Introduction

Colorectal cancer (CRC) continues to be one of the most prevalent causes of cancer-related illness and death globally, with its occurrence increasing in various parts of the world (1). Accumulating evidence has implicated gut microbiota dysbiosis—characterized by altered microbial diversity and composition—in the pathogenesis of CRC (2). The gut microbiota exerts a profound influence on host metabolic processes, immune function, and intestinal barrier integrity, all of which are critical determinants in colorectal carcinogenesis (3, 4). Consequently, interventions targeting the modulation of gut microbial communities have gained increasing attention as potential strategies for CRC prevention and management (5).

Diet is recognized as one of the most influential and modifiable factors shaping the gut microbiota (6). Diets high in prebiotic fibers, polyphenols, fermented foods, whole grains, legumes, fruits, and vegetables foster the growth of beneficial microbes and promote the production of short-chain fatty acids (SCFAs), which have demonstrated anti-inflammatory, immunomodulatory, and antineoplastic properties (7, 8). Conversely, diets rich in ultra-processed foods and pro-inflammatory components have been linked to gut microbial dysbiosis, systemic inflammation, and elevated cancer risk (9, 10). Several dietary indices, such as the Dietary Inflammatory Index (DII) and Mediterranean diet, have been developed and shown to predict inflammation-related cancer risk, including CRC (11–13).

Despite these advances, most prior research has focused on the inflammatory potential of diet or isolated nutrients rather than comprehensive dietary patterns explicitly designed to promote gut microbiota health (14–16). Moreover, few studies have integrated assessments of gut microbial diversity, intestinal permeability, metabolic biomarkers, psychological health, and frailty status in relation to diet and cancer risk (17). This leaves a critical knowledge gap in understanding the multifactorial pathways linking gut microbiota-supportive diets to colorectal carcinogenesis.

To address these gaps, the Dietary Index for Gut Microbiota (DI-GM) is an *a priori* literature-derived dietary score designed to quantify adherence to a microbiota-supportive diet based on known effects of foods on microbial diversity, fermentation capacity, and intestinal barrier integrity (18). Using a case–control design, we investigated (1) the association between DI-GM and CRC risk and (2) the relationships between DI-GM and multidimensional biomarkers—including inflammatory markers, intestinal permeability, metabolic factors, frailty, depression, and sleep quality. This integrative approach provides a more comprehensive understanding of how microbiota-targeted diet as quantified by the DI-GM dietary index may influence CRC risk.

## Methods

### Study design and participants

**Figure 1:**
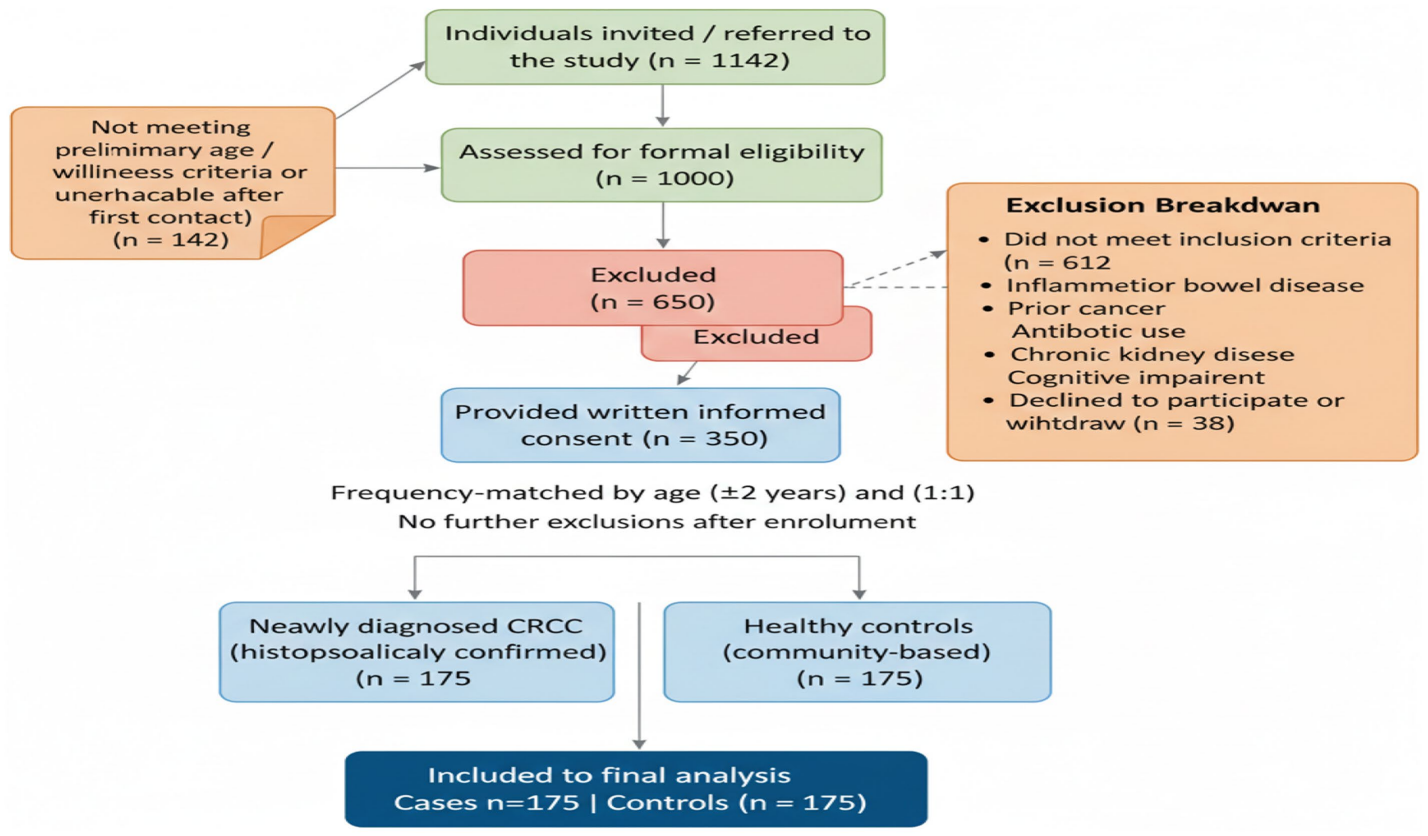
Flowchart illustrating participant recruitment, eligibility assessment, exclusion criteria, and final inclusion of colorectal cancer cases and frequency-matched controls in the case control study.

This case–control study was conducted to examine the association between adherence to a microbiota-targeted diet as quantified by the DI-GM dietary index and CRC risk. A total of 350 adults aged 40–75 years were recruited, including 175 newly diagnosed CRC patients (cases) and 175 healthy controls selected using frequency matching based on age and sex. Cases were confirmed by histopathological diagnosis at Shanxi Cancer Hospital, ensuring all diagnoses met standardized oncological criteria. All CRC patients were enrolled within 2 weeks of histopathological diagnosis and prior to treatment. Controls were recruited from the same geographical area through community health screenings and matched by frequency to cases by age (±2 years) and sex to minimize confounding. Control participants were excluded if they had a history of colorectal cancer or any other malignancy, inflammatory bowel diseases (such as ulcerative colitis or Crohn’s disease), recent gastrointestinal infections or antibiotic use within the past 3 months, chronic autoimmune or systemic inflammatory disorders, or any major chronic diseases that could influence gut microbiota composition or dietary patterns (including, liver cirrhosis, chronic kidney disease, or uncontrolled diabetes). Participants with colorectal polyps, adenomatous lesions, or dysplasia without invasive carcinoma were excluded. Additionally, controls with significant cognitive impairment or inability to provide informed consent were excluded to ensure the reliability of dietary and lifestyle data collection. Exclusion criteria for case group included prior cancer diagnosis, inflammatory bowel disease, recent antibiotic or probiotic use (within 3 months), major chronic illnesses affecting diet or microbiota (e.g., chronic kidney disease, autoimmune disorders), and inability to provide informed consent. Figure 1 provides a step-by-step schematic representation of participant screening, application of inclusion and exclusion criteria, confirmation of newly diagnosed CRC prior to treatment, frequency matching of controls by age and sex, and the final allocation of participants included in the statistical analyses.

### Sample size calculation

Sample size was determined *a priori* using logistic regression power calculations to detect an odds ratio of ≤0.58 for colorectal cancer when comparing the highest versus lowest tertile of the Dietary Index for Gut Microbiota (DI-GM), assuming equal-sized case and control groups, two-sided *α* = 0.05, and 80% power; this yielded a minimum requirement of approximately 150 participants per group. To accommodate potential missing data and to ensure sufficient power for stratified analyses, we enrolled 175 newly diagnosed CRC cases and 175 age- and sex-matched healthy controls (total n = 350). A *post-hoc* power analysis based on the observed fully adjusted odds ratio of 0.32 (95% CI 0.19–0.55) for the highest versus lowest DI-GM tertile confirmed >99.9% power, verifying that the achieved sample size was more than adequate for the primary study objective (13).

### Ethical considerations

The study protocol was approved by the Institutional Review Board of Shanxi Cancer Hospital (Approval No. 20258LL2550). All participants provided written informed consent prior to enrollment, and all procedures adhered strictly to the principles outlined in the Declaration of Helsinki and relevant national guidelines.

### Dietary assessment and DI-GM score calculation

Dietary intake was assessed using three 24-h dietary recalls (two weekdays and one weekend day) conducted on three non-consecutive days including two weekdays and one weekend day to capture typical dietary variation, administered by trained dietitians using standardized multi-pass interview methods. Additionally, a validated semi-quantitative food frequency questionnaire (FFQ) covering habitual intake over the preceding 12 months was administered to capture longer-term dietary patterns (19). Using both recalls and FFQ captured short-term intake variability and habitual long-term diet, reducing measurement error. Nutrient and food group intakes were calculated using comprehensive food composition tables, incorporating local food items and adjustments for preparation methods. The daily nutrient intake for each subject was calculated using the Chinese Food Composition Table. Implausible energy reporters were assessed using the Goldberg cut-off method; none met exclusion criteria (20).

The DI-GM was developed to reflect adherence to a dietary pattern supportive of gut microbiota health (18). The index incorporated intakes of prebiotic fibers, polyphenols, fermented foods, whole grains, legumes, fruits, and vegetables, scored positively based on consumption frequency and quantity. Conversely, consumption of ultra-processed and pro-inflammatory foods—including refined sugars, red and processed meats, and trans fats—was penalized. Each dietary component was weighted according to its established effect on gut microbial modulation in the literature, and individual scores were standardized before summation to generate a composite DI-GM score, with higher scores indicating greater adherence to microbiota-supportive diet.

### Anthropometric and lifestyle data

Measurements followed ISAK guidelines. Weight was measured with a Seca 813 scale and height with a Seca 217 stadiometer. BMI was calculated as kg/m^2^ and classified using WHO cut-offs. Physical activity was assessed using the IPAQ (21).

### Clinical and biochemical measurements

Fasting venous blood samples were collected in the morning following an overnight fast of at least 10 h. Serum and plasma were separated by centrifugation at 3000 rpm for 10 min and stored at −80 °C until analysis. Systemic inflammatory markers, including high-sensitivity C-reactive protein (hs-CRP) and interleukin-6 (IL-6), were quantified using enzyme-linked immunosorbent assays (ELISA) with commercially available kits from Wuhan Fine Biotech Co., Ltd. (Wuhan, China) and R&D Systems China, both validated for clinical research and demonstrating high sensitivity and specificity in previous studies. Additional laboratory parameters, including complete blood count (CBC), fasting insulin, and glucose, were measured using standardized automated analyzers (e.g., Sysmex XN-1000, Roche Cobas 6,000), following rigorous internal and external quality control protocols to ensure accuracy and reproducibility. The ELISA assays were performed according to manufacturers’ protocols, with all samples and standards run in duplicate to minimize analytical variability. Tumor stage at diagnosis was recorded from pathology reports according to the TNM classification (Stage I–IV).

### Frailty assessment

Frailty status was assessed using the modified five-item frailty index (mFI-5), which incorporates comorbidities (e.g., diabetes, hypertension), functional status, and other clinically relevant parameters. The index has been validated in oncologic and general populations and was administered via patient interview and medical record review. Higher scores indicate greater frailty and vulnerability to adverse outcomes (22).

### Psychological assessments

Depressive symptoms were evaluated via the Patient Health Questionnaire-9 (PHQ-9), a widely validated self-administered instrument that assesses the severity of depression over the past 2 weeks. The PHQ-9 is a 9-item questionnaire, with each item rated on a scale from 0 to 3, producing a cumulative score between 0 and 27. Elevated scores reflect increased severity of depressive symptoms, with recognized thresholds categorizing depression as minimal, mild, moderate, or severe. This tool has shown high reliability and validity in various populations, including individuals diagnosed with cancer (23).

Sleep quality was evaluated using the Pittsburgh Sleep Quality Index (PSQI), a generally used self-administered questionnaire designed to assess perceived sleep quality and related disturbances over the preceding month. The PSQI comprises 19 items, organized into 7 components—such as sleep onset latency, efficiency, sleep duration, and daytime impairment. Each component is scored from 0 to 3, contributing to a total global score ranging from 0 to 21, with higher scores reflecting poorer sleep quality. A global score exceeding 5 typically signifies a clinically meaningful sleep disturbance. The PSQI has been extensively validated and is frequently employed in both clinical practice and research, including among cancer patients (24).

### Statistical analysis

All statistical analyses were conducted using Stata version 14. Statistical significance was defined as a two-sided *p*-value < 0.05. Baseline characteristics were summarized as means ± standard deviations for continuous variables and counts with percentages for categorical variables. Normality assumptions were evaluated using the Shapiro–Wilk test. Between-group differences (cases vs. controls) were tested using independent samples t-tests or Mann–Whitney U tests for non-normally distributed variables, and chi-square tests for categorical variables. Participants were categorized into tertiles based on the distribution of DI-GM scores in the total sample. To evaluate the association between DI-GM tertiles and CRC risk, logistic regression analyses were conducted to calculate odds ratios (ORs) and corresponding 95% confidence intervals (CIs), using the lowest tertile as the reference category. Tests for linear trend were conducted by modeling the median value of each tertile as a continuous variable. Linear regression analyses were performed to assess the associations between DI-GM scores (continuous and tertiles) and biomarkers of inflammation, intestinal permeability, metabolic status, psychological health, and frailty. All models included adjustment for relevant covariates, including sociodemographic factors and lifestyle behaviors.

## Results

### Baseline characteristics of participants

Baseline characteristics of contributors, categorized by case and control groups, are summarized in Table 1. The mean age of participants was similar between cancer patients (cases) and healthy controls (59.59 ± 1.09 vs. 59.56 ± 1.10 years; *p* = 0.808). However, the mean DI-GM score was significantly lower in cancer cases compared to controls (7.29 ± 2.7 vs.11.34 ± 2.55; *p* < 0.001), suggesting poorer dietary quality in the case group. Cases also had significantly higher BMI (26.43 ± 4.65 vs. 24.86 ± 1.62 kg/m^2^; *p* < 0.001), greater prevalence of smoking (*p* = 0.036) and alcohol use (*p* = 0.016), and lower levels of physical activity (16.07 ± 7.99 vs. 21.71 ± 7.86 METs; *p* < 0.001).

**Table 1:** Baseline characteristics of participants by case–control status (*n* = 350).

Variable	Case (*n* = 175)	Control (*n* = 175)	*p*-value
Age, years	59.59 ± 1.09	59.56 ± 1.10	0.808
DI-GM Score	7.29 ± 2.70	11.34 ± 2.55	<0.001
BMI, kg/m^2^	26.43 ± 4.65	24.86 ± 1.62	<0.001
Sex, male, n (%)	91 (51)	88 (49)	0.415
Smoking, n (%)	39 (22)	25 (14)	0.036
Alcohol drinking, n (%)	43 (24.5)	26 (15)	0.016
Socio-Economic Status, n (%)			0.519
Low	68 (19.5)	75 (21.5)	
Medium	61 (17.5)	71 (20.3)	
High	39 (11.2)	35 (10)	
Physical activity (total MET), MET	16.07 ± 7.99	21.71 ± 7.86	<0.001
CRP, mg/L	4.84 ± 1.44	2.70 ± 1.37	<0.001
IL-6, pg./mL	4.71 ± 1.34	2.97 ± 1.08	<0.001
Hemoglobin, g/dL	11.78 ± 1.78	13.07 ± 1.80	<0.001
WBC, ×10^3^/μL	7.71 ± 0.96	6.52 ± 0.84	<0.001
NLR	3.35 ± 0.86	1.96 ± 0.91	<0.001
Platelets, ×10^3^/μL	295.79 ± 45.69	259.15 ± 26.12	<0.001
Albumin, g/dL	3.50 ± 0.48	4.03 ± 0.39	<0.001
HbA1c, %	5.66 ± 1.19	5.47 ± 0.56	0.052
PHQ-9, score	11.95 ± 4.42	9.63 ± 4.14	<0.001
PSQI, score	9.90 ± 3.60	8.06 ± 3.43	<0.001
Insulin, μIU/mL	17.29 ± 7.65	12.53 ± 7.50	<0.001
HOMA-IR	4.84 ± 2.23	3.25 ± 2.25	<0.001
Energy, kcal/day	1939.40 ± 242.67	1973.17 ± 255.64	0.207
Fiber, g/day	15.4 ± 6.95	18.42 ± 8.34	0.381
Carbs, g/day	274.69 ± 19.84	276.63 ± 21.21	0.378
Protein, g/day	61.20 ± 13.32	63.09 ± 14.21	0.201
Fat, g/day	66.21 ± 13.81	68.26 ± 14.14	0.172
Systolic BP, mmHg	127.61 ± 8.36	129.43 ± 8.15	0.040
Diastolic BP, mmHg	81.63 ± 4.16	82.38 ± 4.28	0.098
Glucose, mg/dL	91.06 ± 4.32	91.81 ± 4.41	0.110
HDL, mg/dL	45.72 ± 7.01	44.51 ± 6.95	0.105
TG, mg/dL	129.83 ± 4.86	130.14 ± 5.24	0.567
LDL, mg/dL	94.83 ± 6.66	95.83 ± 6.82	0.164
Cholesterol, mg/dL	185.98 ± 11.68	187.78 ± 11.95	0.156

Data are mean ± SD or n (%). *p*-values from independent t-test or chi-square as appropriate. DI-GM, Dietary Index for Gut Microbiota; CRP, C-reactive protein; IL-6, Interleukin 6; FCP, Fecal calprotectin; LBP, Lipopolysaccharide-binding protein; TNF, Tumor necrosis factor-alpha; PHQ-9, Patient Health Questionnaire-9; PSQI, Pittsburgh Sleep Quality Index; HOMA-IR, Homeostatic Model Assessment for Insulin Resistance; MET, Metabolic Equvalent Task.

Markers of systemic inflammation including CRP (4.84 ± 1.44 vs. 2.70 ± 1.37 mg/L; *p* < 0.001) and IL-6 (4.71 ± 1.34 vs. 2.97 ± 1.08 pg./mL; *p* < 0.001) were significantly elevated in the case group.

Furthermore, cases exhibited poorer metabolic and clinical profiles, including lower hemoglobin (11.78 ± 1.78 vs. 13.07 ± 1.80 g/dL; *p* < 0.001), higher WBC counts (7.71 ± 0.96 vs. 6.52 ± 0.84 × 10^3^/μL; *p* < 0.001), elevated NLR (3.35 ± 0.86 vs. 1.96 ± 0.91; *p* < 0.001), higher platelet counts, and reduced albumin levels. Psychological and metabolic indicators such as PHQ-9, PSQI, insulin, and HOMA-IR were also significantly poorer among cancer patients. Dietary intake of macronutrients did not significantly differ between groups.

Among CRC patients, 28 (16.0%) were classified as Stage I, 46 (26.3%) as Stage II, 61 (34.9%) as Stage III, and 40 (22.9%) as Stage IV at diagnosis. DI-GM scores did not differ significantly across tumor stages (*p* = 0.41; Supplementary Table S1).

### Association of DI-GM tertiles with biomarkers

Table 2 shows the distribution of inflammatory, microbial, and psychological biomarkers across tertiles of the DI-GM score, stratified by case–control status. In both groups, higher DI-GM tertiles were associated with significantly lower levels of inflammatory markers (CRP, IL-6, TNF-*α*), intestinal permeability markers (Zonulin, FCP), and psychological distress (PHQ-9, PSQI; *p* < 0.001 for all).

**Table 2:** Associations of dietary index for gut microbiota (DI-GM) across tertiles with biomarkers in case and control groups.

Variable	Tertile 1	Tertile 2	Tertile 3	*p* value^1^	Tertile 1	Tertile 2	Tertile 3	*p* value^1^
	Case group (*n* = 175)				Control group (*n* = 175)			
CRP (mg/L)	5.65 ± 0.82	4.03 ± 1.34	1.90 ± 0.50	<0.001	5.38 ± 0.18	3.16 ± 1.40	1.92 ± 0.15	<0.001
IL-6 (pg/mL)	5.39 ± 1.08	4.01 ± 1.06	2.30 ± 0.50	<0.001	5.14 ± 0.10	3.31 ± 1.07	2.35 ± 0.12	<0.001
Zonulin (ng/mL)	57.1 ± 22.38	57.7 ± 18.26	31.4 ± 0.54	0.006	69.3 ± 0.78	50.3 ± 20.17	39.0 ± 16.00	<0.001
FCP (μg/g)	97.0 ± 39.54	94.7 ± 36.43	41.1 ± 1.07	0.001	119.4 ± 2.38	80.3 ± 40.30	58.5 ± 32.39	<0.001
TNF-α (pg/mL)	22.5 ± 8.96	20.4 ± 7.24	9.43 ± 0.53	<0.001	24.9 ± 0.71	17.7 ± 7.95	13.1 ± 6.09	<0.001
PHQ-9	12.4 ± 4.68	12.0 ± 3.92	6.57 ± 0.53	<0.001	14.6 ± 0.49	10.6 ± 4.45	8.15 ± 3.42	<0.001
PSQI	10.3 ± 3.86	9.8 ± 3.12	5.57 ± 0.53	0.003	12.1 ± 0.68	8.9 ± 3.75	6.8 ± 2.82	<0.001
Frailty Index	2.75 ± 0.16	1.98 ± 0.60	1.00 ± 0.2	<0.001	–	–	–	–
CEA (ng/mL)	4.65 ± 2.99	5.15 ± 3.18	6.20 ± 2.85	0.314	–	–	–	–
CA19-9 (U/mL)	29.9 ± 23.58	34.2 ± 25.68	44.0 ± 22.32	0.232	–	–	–	–

Data presented as mean ± standard deviation. DI-GM, Dietary Index for Gut Microbiota; FCP, Fecal Calprotectin; IL-6, Interleukin 6; TNF-α, Tumor Necrosis Factor-alpha; PHQ-9, Patient Health Questionnaire-9; PSQI, Pittsburgh Sleep Quality Index; CEA, Carcinoembryonic Antigen; CA19-9, Carbohydrate Antigen 19–9. Frailty was assessed using the modified five-item frailty index (mFI-5). Tertiles were determined separately in case and control groups. “–” indicates data not collected, not applicable, or not analyzed for that group.

1 *p*-values are from ANOVA across DI-GM tertiles in each group separately.

Among cancer patients, CRP levels decreased progressively from 5.65 ± 0.82 mg/L in the lowest DI-GM tertile to 1.90 ± 0.5 mg/L in the highest (*p* < 0.001). A similar trend was observed for IL-6 (5.39 ± 1.08 to 2.30 ± 0.5 pg./mL), and TNF-α. Notably, the frailty index was significantly lower in the highest tertile compared to the lowest (2.75 ± 0.16 vs. 1.00 0.5; *p* < 0.001).

Comparable trends were observed in the control group. For example, IL-6 decreased from 5.14 ± 0.10 pg./mL in the lowest tertile to 2.35 ± 0.12 pg./mL in the highest (*p* < 0.001), and PHQ-9 scores declined from 14.6 ± 0.49 to 8.15 ± 3.42 (*p* < 0.001). This indicates a robust dose–response relationship between DI-GM and multiple health indicators in both groups.

### Multivariate associations between DI-GM and clinical biomarkers

As shown in Table 3, multivariable linear regression analysis among cancer patients revealed that higher DI-GM scores were strongly and independently associated with favorable inflammatory and clinical profiles. In the fully adjusted model (Model 3), each unit increase in DI-GM was associated with significant reductions in CRP (B = −1.90; 95% CI: −2.35, −1.88; *p* < 0.001), IL-6 (B = −2.20; 95% CI: −2.23, −2.18; *p* < 0.001), TNF-*α* (B = −0.27; 95% CI: −0.30, −0.23; *p* < 0.001), and Zonulin (B = −0.11; 95% CI: −0.12, −0.09; *p* < 0.001).

**Table 3:** Association of DIGM with inflammatory and clinical markers in patients with cancer (*n* = 175)†.

Variable	Model 1a: B (95% CI)	*p*-value*	Model 2b: B (95% CI)	*p*-value*	Model 3c: B (95% CI)	*p*-value*
CRP (mg/dL)	−1.87 (−1.89, −1.85)	<0.001	−1.89 (−1.91, −1.88)	<0.001	−1.90 (−2.35, −1.88)	<0.001
IL-6 (pg/mL)	−2.19 (−2.23, −2.16)	<0.001	−2.20 (−2.23, −2.18)	<0.001	−2.20 (−2.23, −2.18)	<0.001
TNF-α (pg/mL)	−0.26 (−0.30, −0.23)	<0.001	−0.26 (−0.30, −0.23)	<0.001	−0.27 (−0.30, −0.23)	<0.001
Zonulin (ng/mL)	−0.08 (−0.09, −0.06)	<0.001	−0.10 (−0.12, −0.08)	<0.001	−0.11 (−0.12, −0.09)	<0.001
Frailty Index	−3.91 (−4.21, −3.62)	<0.001	−4.25 (−4.38, −4.12)	<0.001	−4.27 (−4.40, −4.14)	<0.001
PHQ-9 (Score)	−0.37 (−0.44, −0.30)	<0.001	−0.47 (−0.56, −0.39)	<0.001	−0.48 (−0.57, −0.40)	<0.001

†Analysis limited to cancer patients. *p-values based on linear regression. Model 1a: Unadjusted. Model 2b: Adjusted for age, sex, and BMI. Model 3c: Adjusted for Model 2 + total energy intake (kcal).

Additionally, higher DI-GM was significantly associated with lower frailty scores (B = −4.27; 95% CI: −4.40, −4.14; *p* < 0.001) and PHQ-9 scores (B = −0.48; 95% CI: −0.57, −0.40; *p* < 0.001), independent of demographic and dietary confounders.

### Association between DI-GM score and Cancer risk

Table 4 presents the association between DI-GM tertiles and colorectal cancer risk. In the crude model, participants in the highest DI-GM tertile had 62% lower odds of CRC compared with those in the lowest tertile (OR = 0.38, 95% CI 0.23–0.63). After adjustment for age, sex, and BMI (Model 1), the OR was 0.34 (95% CI 0.20–0.58). In the fully adjusted model (Model 2) that additionally controlled for total energy intake, carbohydrate intake, and protein intake, individuals in the highest tertile showed 68% lower odds of colorectal cancer (OR = 0.32, 95% CI 0.19–0.55) compared with the lowest tertile. A significant dose–response relationship was observed across tertiles (P for trend < 0.001).

**Table 4:** Association between DI-GM tertiles and cancer risk.

Variable	OR (95% CI)—crude model	Model 1a	Model 2a	P for trend*
DI-GM tertiles				<0.001
Tertile 1 (Low)	Reference	Reference	Reference	
Tertile 2 (Moderate)	0.55 (0.34–0.89)	0.52 (0.31–0.87)	0.50 (0.30–0.84)	
Tertile 3 (High)	0.38 (0.23–0.63)	0.34 (0.20–0.58)	0.32 (0.19–0.55)	

*P for trend: Based on logistic regression treating DI-GM tertile as ordinal variable. Crude Model: Unadjusted. Model 1a: Adjusted for age, sex, and BMI. Model 2a: Model 1 plus total energy intake, carbohydrate, and protein intake.

## Discussion

This study demonstrates that adherence to a gut microbiota-supportive dietary pattern—as quantified by the DI-GM—is strongly associated with reduced CRC risk and with favorable inflammatory, microbial, metabolic, psychological, and frailty profiles. The magnitude of the association (68% lower odds for highest vs. lowest tertiles) is comparable to or stronger than associations reported for the Mediterranean and other plant-forward diets.

A recent large-scale prospective cohort study by Li et al. reported an inverse association between the DI-GM and the risk of gastrointestinal cancers (25). While that study provided important epidemiological evidence, our investigation differs substantially in three aspects. First, we focused exclusively on colorectal cancer rather than gastrointestinal cancers as a group, allowing disease-specific interpretation. Second, we incorporated detailed inflammatory, microbial, intestinal permeability, psychological, and frailty assessments in newly diagnosed patients prior to treatment, providing mechanistic insight beyond risk association. These elements extend prior findings and highlight the multi-system relevance of microbiota-supportive diets in CRC. Our findings are consistent with emerging evidence that suggests diet-mediated modulation of the gut microbiota plays a critical role in cancer prevention and overall systemic health (11–13). Specifically, individuals with higher DI-GM scores demonstrated significantly lower levels of pro-inflammatory biomarkers, including CRP, IL-6, TNF-*α*, and Zonulin. This finding is particularly important as it indicates that dietary patterns can influence systemic inflammation, which is a known risk factor for various chronic diseases, including cancer (26). This observation aligns with prior research demonstrating that diets rich in dietary fiber and polyphenols can effectively reduce inflammation through the production of short-chain fatty acids (SCFAs) (27). SCFAs inhibit the nuclear factor kappa-light-chain-enhancer of activated B cells (NF-κB) pathway, which is instrumental in reducing pro-inflammatory cytokines (28, 29).

Psychological outcomes, such as depression and sleep quality, also exhibited a significant inverse relationship with DI-GM scores. This finding is consistent with the proposed mechanisms of the gut brain axis, suggesting that gut health can influence mental health outcomes (30, 31). This connection highlights the multifaceted nature of health, where dietary choices not only affect physical health but also mental well-being (26, 32, 33). Our findings resonate with previous studies that have linked dietary patterns that promote microbial balance to diminished depressive symptoms. This effect may occur through the modulation of tryptophan metabolism, immune signaling pathways, and the vagus nerve (34). Additionally, the inverse association between DI-GM scores and the frailty index further indicates that interactions between diet and microbiota may significantly impact physical resilience in cancer patients. This suggests that dietary interventions could play a crucial role in enhancing the overall quality of life for cancer patients by improving both their physical and psychological health.

Importantly, in this case–control study of 350 Chinese adults, higher adherence to a gut microbiota-supportive dietary pattern, as reflected by higher DI-GM scores, was independently associated with substantially lower odds of colorectal cancer. Participants in the highest tertile of DI-GM had 68% lower odds of CRC (fully adjusted OR = 0.32, 95% CI 0.19–0.55; P for trend < 0.001) compared with those in the lowest tertile. This association remained robust after adjustment for age, sex, BMI, total energy intake, and macronutrient composition, and represents one of the strongest protective dietary associations reported for colorectal cancer to date, comparable in magnitude to high adherence to the Mediterranean or plant-based dietary patterns in previous meta-analyses. To our knowledge, the first CRC-specific study integrating to demonstrate such a pronounced inverse association between a microbiota-focused dietary index and cancer risk in a human population. No association was observed between DI-GM and tumor stage, suggesting that microbiota-supportive dietary habits may be more strongly related to CRC risk than to disease severity at the time of diagnosis. These findings suggest that dietary patterns that promote a healthy gut microbiota may serve as a modifiable protective factor in cancer prevention and survivorship strategies. Our results are consistent with prior research showing that diets rich in fiber, polyphenols, and fermented foods—such as the Mediterranean or MIND diets—are linked to a lower risk of gastrointestinal malignancies (11–13). Furthermore, several recent studies (2024–2025) reinforce the protective effects of high-quality, anti-inflammatory, or microbiota-supportive dietary patterns against CRC and gastrointestinal (GI) cancers, while contrasting them with risks from Western-style or pro-inflammatory diets. For example, a 2025 NHANES analysis showed higher adherence to the Healthy Eating Index-2020 (HEI-2020) and alternative Mediterranean Diet (aMED) scores linked to reduce GI cancer risk, highlighting overall diet quality’s role in prevention (35). A 2022 EPIC-Spain cohort found suggestive increased CRC risk with Western patterns (especially in females and rectal subsite) and protective trends with Mediterranean adherence (particularly early follow-up and distal colon), with limited Prudent pattern effects (36). The 2024 review by Adolph and Tilg connects Western diets to gut microbial dysbiosis, chronic inflammation, and elevated risks of malignancies including CRC, emphasizing the harms of processed foods and low microbiota-supportive elements (37).

Prospective evidence using DI-GM (analogous to ours) further supports microbiota-targeted approaches: a 2025 UK Biobank gene-diet study (*n* = 178,148; median 13.47-year follow-up) reported higher DI-GM associated with lower GI cancer risk (HR 0.84 for CRC in highest vs. lowest category; HR 0.83 overall GI cancer), with stronger effects in lower genetic risk groups and significant DI-GM–genetic risk interaction (25). Broader systematic reviews, such as the Global Cancer Update Program (CUP Global, 2025), graded limited-suggestive evidence for lower CRC risk with higher alignment to *a priori* patterns like Mediterranean, healthful plant-based, Healthy Eating Index (HEI)/alternate HEI, and DASH, versus strong-probable increased risk from hyperinsulinemic (EDIH) or proinflammatory (EDIP) patterns (especially colon). A large prospective Study (*n* = 542,778 women; 12,251 CRC cases over ~16.6 years;) identified strong inverse associations with calcium intake (RR 0.83 per 300 mg/day) and dairy-related factors, alongside positive links to red/processed meat (RR 1.08 per 30 g/day) and alcohol, reinforcing calcium’s protective role (largely driving dairy benefits) and risks from less favorable patterns (38).

However, unlike previous studies, the DI-GM specifically integrates dietary components known to modulate microbial diversity and function, thereby offering a more targeted approach to gut-mediated cancer prevention. Mechanistically, the protective effect of a high DI-GM score may be explained, in part, by enhanced microbial production of short-chain fatty acids (SCFAs), such as butyrate and propionate, which have been shown to exert anti-inflammatory, anti-proliferative, and epigenetic effects on colonocytes (39, 40). In addition, a higher DI-GM score may contribute to better gut barrier function and immune regulation—both critical in mitigating tumorigenesis—potentially through modulation of the gut microbiota.

However, it is essential to acknowledge certain limitations of this study. First, the case–control design inherently carries the risk of reverse causation and recall bias, which must be considered. Although we used multiple 24-h recalls and FFQs to minimize recall bias, the diagnosis of CRC may influence patients’ recall of past dietary habits. Because of the cross-sectional case–control design, temporal relationships and mediation pathways cannot be established. Second, despite adjusting for multiple demographic and lifestyle factors, we cannot completely rule out residual confounding from unmeasured or unknown variables, such as socioeconomic status, genetic predisposition, or specific medication use (e.g., chronic PPI use) that could influence both diet and CRC risk. Additionally, we cannot establish a causal link. Finally, dietary intake was self-reported, which is susceptible to recall bias, potentially affecting the accuracy of our findings. Addressing these limitations in future research will be crucial for validating our findings and enhancing the reliability of dietary recommendations. Future longitudinal and intervention studies are needed to formally evaluate potential biological pathways linking microbiota-targeted dietary patterns and colorectal carcinogenesis.

## Conclusion

Higher adherence to a gut microbiota-supportive dietary pattern—reflected by higher DI-GM scores—is associated with significantly lower CRC risk, reduced systemic inflammation, enhanced gut barrier integrity, and better psychosocial outcomes. These findings underscore the potential of microbiota-focused dietary interventions as feasible, modifiable strategies to reduce CRC risk and improve overall health. Prospective cohort studies and randomized dietary trials are warranted to establish causality and inform clinical translation.

## Data Availability

The raw data supporting the conclusions of this article will be made available by the authors, without undue reservation.
