# Supplementary material for: Dietary index for gut microbiota is inversely associated with colorectal cancer risk: a case–control study
Source: Front Nutr. 2026 Mar 6;13:1762018. doi: 10.3389/fnut.2026.1762018 (PMC13002377; doi:10.3389/fnut.2026.1762018)
Supplement: Supplementary file 1 [file Table_1.docx]

| Table S.1. DI-GM Score According to Tumor Stage Among CRC Patients (n = 175) | | |
| --- | --- | --- |
| Tumor Stage | n | DI-GM score (mean ± SD) |
| Stage I | 28 | 7.52 ± 2.61 |
| Stage II | 46 | 7.31 ± 2.73 |
| Stage III | 61 | 7.18 ± 2.66 |
| Stage IV | 40 | 7.05 ± 2.74 |
| P-value (ANOVA) |  | 0.41 |
| Data are presented as mean ± standard deviation. P-value derived from one-way ANOVA across tumor stages. | | |
